# Autoprocessing of human immunodeficiency virus type 1 protease miniprecursor fusions in mammalian cells

**DOI:** 10.1186/1742-6405-7-27

**Published:** 2010-07-28

**Authors:** Liangqun Huang, Chaoping Chen

**Affiliations:** 1 Department of Biochemistry and Molecular Biology, Colorado State University, Fort Collins, Colorado, USA

## Abstract

**Background:**

HIV protease (PR) is a virus-encoded aspartic protease that is essential for viral replication and infectivity. The fully active and mature dimeric protease is released from the Gag-Pol polyprotein as a result of precursor autoprocessing.

**Results:**

We here describe a simple model system to directly examine HIV protease autoprocessing in transfected mammalian cells. A fusion precursor was engineered encoding GST fused to a well-characterized miniprecursor, consisting of the mature protease along with its upstream transframe region (TFR), and small peptide epitopes to facilitate detection of the precursor substrate and autoprocessing products. In HEK 293T cells, the resulting chimeric precursor undergoes effective autoprocessing, producing mature protease that is rapidly degraded likely via autoproteolysis. The known protease inhibitors Darunavir and Indinavir suppressed both precursor autoprocessing and autoproteolysis in a dose-dependent manner. Protease mutations that inhibit Gag processing as characterized using proviruses also reduced autoprocessing efficiency when they were introduced to the fusion precursor. Interestingly, autoprocessing of the fusion precursor requires neither the full proteolytic activity nor the majority of the N-terminal TFR region.

**Conclusions:**

We suggest that the fusion precursors provide a useful system to study protease autoprocessing in mammalian cells, and may be further developed for screening of new drugs targeting HIV protease autoprocessing.

## Background

Human immunodeficiency virus 1 (HIV-1) is the causative pathogen of AIDS. The HIV protease is a virus-encoded enzyme absolutely required for virus propagation and infectivity. In the HIV infected cell, unspliced genomic RNA serves as mRNA for the synthesis of Gag and Gag-Pol polyproteins [1,2]. As part of the Gag-Pol polyprotein, the HIV protease is flanked at the N-terminus by a transframe region (TFR) and at the C-terminus by the reverse transcriptase [3,4]. The embedded protease has intrinsic but limited proteolytic activity [5,6] and the full activity is associated with the mature protease following its liberation from the precursor. Production of mature protease appears to be catalyzed by the Gag-Pol precursor itself serving as both the substrate and enzyme, thus the process is defined as protease autoprocessing [3] although it remains unclear whether the initial cleavage is intra- or inter-molecular [7,8]. The mature protease contains 99 amino acid residues and is a member of the aspartyl protease family [3,4,9]. It exists as stable homodimers (*K*_d _< 5 nM) and the catalytic site is formed at the dimer interface by two aspartic acids, one from each monomer, that are required for proteolytic activity. Alteration of D25 to either asparagine or alanine abolish protease activity *in vitro *and *in vivo *[3,10-12]. Because of the requirement for two aspartate residues that are at the dimer interface, it is believed that protease precursor dimerization is essential for formation of the catalytic site to initiate protease autoprocessing [3].

HIV protease cleaves multiple sites in the Gag and Gag-Pol polyproteins [3]. The cleavage efficiency at each recognition site varies likely due to the diversity of substrate sequences [13]. Some of these sites, such as the MA/CA and the p2/NC sites, can be cleaved by both precursor and mature proteases [6,13], and peptides containing these sites have been used as standard substrates for examination of protease activity *in vitro*. In contrast, other recognition sites require the fully active mature protease. For example, cleavage of p25 (CA-p2) at the CA/p2 site, which releases p24 (CA), has been shown to require the fully active mature protease. In fact, the amount of p24 (CA) relative to p25 (CA-p2) and other p24-containing proteins such as the full length Gag polyprotein in the released virions, *i.e*. Gag processing efficiency, has been used as an indirect measurement to reflect mature protease activity and/or protease autoprocessing efficiency [14]. Effective cleavage of all these sites following a defined sequence is essential for the production of infectious progeny virions. Mutations that alter the time of processing, the order in which the sites are cleaved, or that produce an incorrect cleavage at any individual site, cause the release of aberrant virions that are significantly less infectious [15-18]. Because HIV protease plays a critical role in viral infectivity, protease inhibitors targeting the catalytic site have been routinely used in combination with inhibitors targeting other viral components in antiretroviral therapy (ART).

In contrast to the well known function of the HIV protease, the molecular and cellular mechanisms mediating precursor autoprocessing remains largely illusive. Between the two cleavage sites that lead to liberation of the mature protease, the C-terminal cleavage seems to have less of an impact on the regulation of autoprocessing as mutations blocking this cleavage have no significant influence on protease activity or Gag processing in transfected mammalian cells [19,20]. In contrast, mutations blocking N-terminal cleavage abolish Gag processing and lead to loss of viral infectivity [21,22], suggesting that N-terminal cleavage plays an important role in regulating autoprocessing. Consistent with this, a miniprecursor comprised of a slightly modified mature protease plus the upstream TFR has been utilized as a model system to study protease autoprocessing [3,23]. When expressed in *E. coli*, the miniprecursor is predominantly associated with inclusion bodies and is therefore purified under denaturing conditions and refolded *in vitro*. Structural and functional analyses of the resulting miniprecursor have demonstrated that cleavage at the N-terminus of the protease is concomitant with the formation of a stable dimer and the appearance of catalytic activity [3]. Another approach to assess autoprocessing is to use proviruses that carry various protease mutations; however, it has been difficult to directly detect autoprocessing intermediates associated with transfected or infected cells. Because of this limitation, proteolytic cleavage of the Gag polyprotein has been measured as an indirect readout of autoprocessing efficiency and/or protease activity.

In order to further define viral and/or cellular determinants that regulate HIV protease autoprocessing, we recently reported a GST-miniprecursor fusion that undergoes autoprocessing in *E. coli *[24]. GST was chosen as a fusion tag to increase protein solubility and facilitate precursor dimerization. The reported GST-TFR-PR contains two natural cleavage sites: one at the N-terminus of TFR (referred to as the distal site) and the other between TFR and PR (the proximal site). In the present study, a similar fusion construct was engineered for mammalian expression to examine protease autoprocessing in transfected mammalian cells. Autoprocessing of the fusion precursors carrying protease mutations that were previously characterized with provirus constructs was examined to evaluate utility of the system. We demonstrate that the GST-miniprecursor fusions mirror phenotypes described in other model systems and therefore provide a simple system for further analysis of protease autoprocessing.

## Results

### GST fused protease miniprecursors undergo autoprocessing in E. coli and HEK293T cells

**Figure 1:**
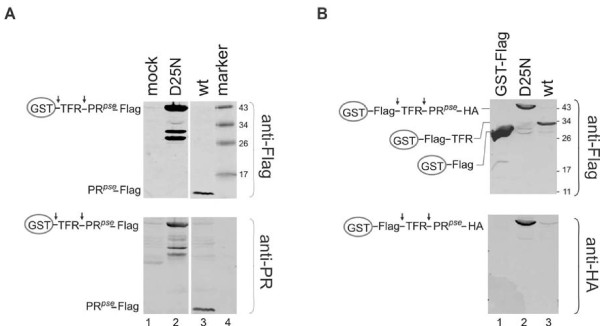
**Autoprocessing of GST- miniprecursor fusions in *E. coli *and HEK 293T cells**. A. Bacteria *E. coli *BL21(DE3) bearing pGEX-3X derived plasmids encoding the indicated miniprecursor construct were induced with 40 μM of IPTG to express fusion proteins. Total cell lysates were subjected to 12% SDS-PAGE and western blotting using monoclonal mouse anti-Flag and polyclonal rabbit anti-PR primary antibodies and IR700 goat anti-mouse and IR800 goat anti-rabbit secondary antibodies. Images of both channels are presented. Samples were run on the same gel but lanes were re-arranged for presentation. Schematic diagrams of the full-length fusion precursor and processing products are indicated at left. **B**. HEK293T cells were transfected with pEBG-derived plasmids expressing the indicated fusion protein using the calcium phosphate method. Post-nuclear cell lysates were prepared at 40 h post-transfection and analyzed by 12% SDS-PAGE and western blotting. Aliquots (~ 20 μL) of each sample were examined in parallel with either monoclonal mouse anti-Flag or anti-HA primary antibody and IR800 goat anti-mouse secondary antibody. Molecular mass markers (kDa) are indicated at right.

We previously reported that a miniprecursor fusion (GST-TFR-PR*^pse^*-Flag) exhibited autoprocessing in *E. coli*, and we were able to isolate Flag-tagged mature protease from whole cell lysates using anti-Flag antibody [24]. Here, we demonstrate that the mature protease is the predominant product in *E. coli *whole cell lysates as detected with either anti-Flag or anti-PR antibody (Figure 1A lane 3). It is unlikely that the cleavage reactions were catalyzed by a cellular protease because catalytic site mutation (D25N) ablated autoprocessing resulting in the full-length fusion precursor as the major band (Figure 1A lane 2). We also observed two bands that are smaller than the full length fusion precursor in the D25N mutant. The fact that both fragments were reactive to anti-PR and anti-Flag suggested that they were likely produced as a result of proteolytic cleavage in the GST domain. These cleavages appear characteristic with fusion precursors with inactive (D25N) or reduced (H69E) protease activities as reported previously [24], but are beyond the expected cleavage sites essential for protease autoprocessing. We did not pursue this further as it seems unrelated to protease autoprocessing

We next constructed a mammalian expression plasmid to evaluate autoprocessing of GST-miniprecursor fusion in mammalian cells. The rabbit anti-PR used for protease detection in *E. coli *lysates failed to distinguish positive signal from background noise in 293T lysates (data not shown). To facilitate detection of processing intermediates, we engineered the expression plasmid to have a Flag tag between the GST and the TFR, and a HA tag at the C-terminus of the protease. The resulting fusion precursor (GST-Flag-TFR-PR*^pse^*-HA) exhibited effective autoprocessing in transfected HEK 293T cells as indicated by the disappearance of the full length precursor and appearance of a processing product (GST-Flag-TFR) (Figure 1B lane 3). Like in *E. coli*, autoprocessing was dependent on active HIV protease because the mutant precursor (D25N) with a deficient catalytic site exhibited little or no autoprocessing (Figure 1B lane 2). Unlike in *E. coli*, where the mature protease is detectable, the HA-tagged mature protease was not detected by the HA antibody even though the antibody successfully identified the full-length precursor. We interpreted that the mature protease was rapidly degraded in transfected mammalian cells as a result of autoproteolysis that is characteristic of HIV PR [25]. Interestingly, only the proximal site of the pseudo wild-type protease miniprecursor was cleaved, releasing GST-Flag-TFR; no GST-Flag was produced suggesting the distal site was not cleaved. Nevertheless, our data demonstrated that the GST-miniprecursor fusions are competent for autoprocessing in mammalian cells.

### Darunavir and Indinavir inhibit fusion precursor autoprocessing in transfected 293T cells

**Figure 2:**
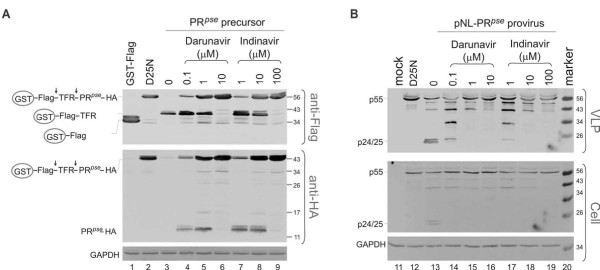
**Known protease inhibitors block protease autoprocessing**. **A**. HEK293T cells transfected with the indicated pEBG construct were incubated with or without protease inhibitors at increasing concentrations. Darunavir: 0.1 μM, 1 μM and 10 μM; Indinavir: 1 μM, 10 μM and 100 μM. Post-nuclear cell lysates were prepared at 40 h post-transfection and aliquots (~20 μL) of each sample were analyzed in parallel using monoclonal mouse anti-Flag, anti-HA, anti-GAPDH primary antibodies and IR800 goat anti-mouse secondary antibody. Schematic diagrams of the full length fusion precursor and processing products are indicated at left. Molecular mass markers (kDa) are indicated at right. **B**. HEK293T cells that were transfected with NL4-3-derived proviruses encoding the indicated proteases were incubated with or without protease inhibitors at the same concentrations as in panel A. Post-nuclear cell lysates (Cell) and VLP particles (VLP) were prepared as described (Materials and Methods) and subjected to western blot analysis using monoclonal mouse anti-p24. The full length Gag polyprotein (p55) and p24/p25 doublet are indicated at left.

To further examine autoprocessing specificity, we next tested whether known HIV protease inhibitors suppress autoprocessing. In the absence of inhibitors, the pseudo wild type precursor fusion effectively underwent autoprocessing; almost no full-length precursor was detected (Figure 2 lane 3). In contrast, the D25N mutant demonstrated the full length precursor as the major product that was detected by both anti-Flag and anti-HA antibodies. In the presence of cell-permeable Darunavir and Indinavir [26,27], precursor autoprocessing was inhibited in a dose-dependent manner, as indicated by the appearance of increasing amounts of full length precursor. In addition, HA-tagged mature protease became detectable in the presence of low concentrations of protease inhibitor. This data suggested that reduced protease activity hindered degradation of the mature protease whereas the fully active mature protease is prone to complete degradation.

We also examined inhibitor effects on NL4-3-derived proviral constructs for comparison with the GST fusion miniprecursor system (Figure 2B). A mouse monoclonal p24 antibody was used to detect p24 and other p24-containing proteins such as p55. Steady-state levels of the full-length Gag polyprotein (p55) in transfected cells were very similar in the presence or absence of inhibitors (Figure 2B, bottom two panels). The amounts of p24 in virus-like particles (VLPs) released into the culture medium were examined as an indirect measurement of protease activity and/or autoprocessing efficiency[14]. The top panel of Figure 2B demonstrated that p24 protein was easily detectable in VLPs produced by the pseudo wild-type HIV protease construct (Figure 2B lane 13), whereas VLPs produced by the D25N mutant contained only the full length Gag (Figure 2B lane 12). In the presence of protease inhibitors, Gag processing was impeded in a dose-dependent manner. At low concentrations of inhibitors, Gag polyprotein was partially processed as indicated by the presence of some processing intermediates (Figure 2B lanes 14 & 17). It should be noted, however, that very little or no p24 was detected even at low concentrations of inhibitor, confirming that p24 production strictly requires the fully active mature protease. In the presence of high concentrations of inhibitors, the full length Gag polyprotein became the predominant product in the released VLPs, indicating a complete lack of protease activity. Our data indicated that Gag processing in VLP qualitatively correlated with autoprocessing of the GST-fused precursors in transfected mammalian cells. Partial inhibition of protease activity completely prevented production of p24, but only partially blocked the autoprocessing of the GST-fusion precursors.

### Autoprocessing of mutant fusion precursors in transfected 293T cells

**Figure 3:**
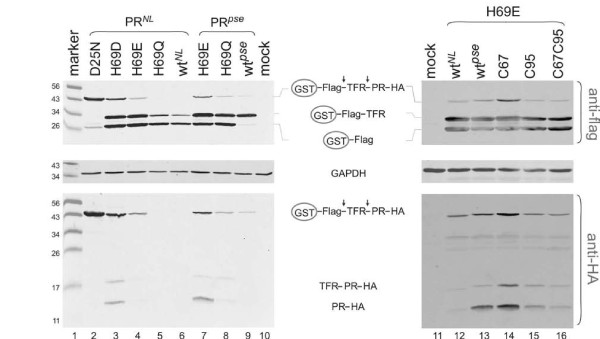
**Differential effects of H69 mutations on protease autoprocessing**. pEBG-derived plasmids expressing the indicated fusion proteins were transfected into HEK293T cells using calcium phosphate and post-nuclear cell lysates were prepared at 40 h post transfection. Aliquots (~20 μL) of each sample were subjected to western blotting in parallel using monoclonal mouse anti-Flag or anti-HA primary antibodies and IR800 goat anti-mouse second antibody. The full-length fusion precursor and processing products are indicated in the middle; molecular mass markers (kDa) are indicated at left.

We next constructed precursor fusions carrying previously characterized mutations to examine whether the precursor fusions would reproduce previous observations in transfected 293T cells. First, H69 mutations in the context of either the pseudo wild type (PR*^pse^*) or the NL4-3-derived (PR*^NL^*) protease backbone were analyzed (Figure 3, left). H69 is a surface residue on the mature protease, but we recently reported that alterations of H69 modulate precursor structures and thus influence protease autoprocessing and the subsequent Gag processing [14,24]. For example, PR*^pse ^*H69E was defective for Gag processing in VLPs produced from cells that were transfected with PR*^pse ^*H69E proviral DNA, whereas H69Q had minimal impact [24]. Here, we also found reduced autoprocessing in cells transfected with the PR*^pse ^*H69E fusion precursor as indicated by the accumulation of the full-length precursor compared to the wild type (PR*^pse^*) and H69Q controls (Figure 3 lane 7-9). In addition, the wild type PR*^pse ^*mature protease was undetectable but HA-tagged mature H69E protease was identified in the cell lysate. Because the H69E pseudo wild type protease has reduced proteolytic activity [24], this indicated once again that inhibition of protease activity slows down protease degradation, consistent with the detection of mature protease in the presence of inhibitors.

Using provirus as a test model we recently demonstrated that H69 mutations in the context of the pseudo wild type (PR*^pse^*) or NL4-3-derived (PR*^NL^*) proteases exert different effects on protease autoprocessing and subsequent Gag processing efficiency. For example, H69E mutation abolished Gag processing in the PR*^pse ^*backbone, but only showed mild reduction in Gag processing in the PR*^NL ^*backbone; the PR*^NL ^*H69D displayed a Gag processing phenotype similar to PR*^pse ^*H69E [14]. With the mammalian GST fusion expression system, we observed similar results, as indicated by the amount of full length precursor remaining in the lysate (Figure 3, left panel). Furthermore, HA-tagged mature proteases containing mutations that significantly reduced Gag processing in the proviral system were also detected in the mammalian expression system (Figure 3, bottom, lanes 3 and 7), suggesting a contribution of reduced protease activity to Gag processing deficiency. There are six point mutations of amino acid between PR*^pse ^*and PR*^NL^*. We recently reported that the inhibitory effect of PR*^pse ^*H69E mutation on Gag processing efficiency is hampered when the same mutation is placed into the PR*^NL ^*backbone and C95 is the primary contributing residue out of the six variations between PR*^pse ^*and PR*^NL ^*[14]. Using the GST fusion precursors, we also observed that PR*^NL ^*H69E had higher protease activity than PR*^pse ^*H69E as indicated by reduced amount of the full-length precursor and no detection of HA-tagged mature protease (Figure 3 lane 4 vs lane 7). PR*^pse ^*H69E/A95C mutation showed autoprocessing activity similar to PR*^NL ^*H69E (lane 12 vs lane 15), consistent with the previous report that C95 residue suppressed the inhibitory effect of H69E in the pseudo wild type backbone. Collectively, our data suggest that mutant phenotypes obtained with other model systems are reproducible with the GST-precursor fusions expressed in mammalian cells and that reduced Gag processing qualitatively correlates with reduced protease activity.

The anti-Flag antibody revealed additional information of protease autoprocessing. We observed moderate amounts of autoprocessing products, such as GST-Flag and GST-Flag-TFR, in all cell lysates except for the D25N negative control, suggesting autoprocessing occurred even with the mutant proteases such as PR*^NL ^*H69D and PR*^pse ^*H69E. Another interesting observation was differential recognition of the proximal and distal cleavage sites. The pseudo wild type protease preferentially cut at the proximal site, directly releasing the mature protease as the only product, whereas the NL4-3-derived protease cut both sites, with a slight preference to the distal site (Figure 3 lane 6). Alteration of H69 in the PR*^pse ^*backbone also changed cleavage preference, resulting release of two GST-containing processing products in PR*^pse ^*H69 mutants.

**Figure 4:**
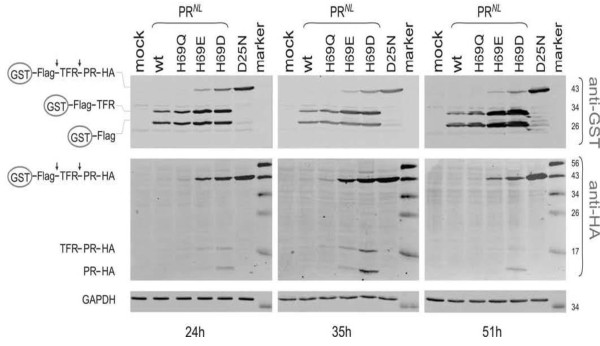
**Temporal analysis of protease autoprocessing**. pEBG-derived plasmids expressing the indicated fusion precursors were transfected into HEK293T cells using calcium phosphate. Post-nuclear cell lysates were prepared at the indicated times post transfection and subjected to western blot analysis using polyclonal rabbit anti-GST (top) and mouse anti-HA (middle) primary antibodies, and IR800 goat anti-rabbit and IR700 goat anti-mouse secondary antibodies. The same blot was stripped and analyzed using mouse anti-GAPDH (bottom) as a loading control. The full length fusion precursor and processing products are indicated at left; molecular mass markers (kDa) are indicated at right.

To further study autoprocessing dynamics, we examined steady state levels of protease autoprocessing products at different time points (Figure 4). At 24, 35 and 51 hours post-transfection, the overall distribution pattern of precursor and cleavage products was very similar to that observed at 40 h post-transfection (Figure 3). For active proteases (wt and H69Q), neither the full length precursor nor mature protease was detectable at any time point examined. This suggested a rapid disappearance of both the precursor substrate and the mature protease product over the time course that was examined. The other two autoprocessing products, GST-Flag-TFR and GST-Flag, demonstrated a slight accumulation over time, indicating that they are more stable than the mature protease. The inactive D25N protease also displayed a slight accumulation of the full-length precursor over time. For mutant proteases H69E and H69D, accumulation of HA-tagged mature protease was minimal and transient at 35 h post-transfection and diminished at 51 h post-transfection, indicating that degradation of these mutant proteases was slower, but was eventually complete when production of the precursor decreased over time. Our data indicated that protease autoprocessing occurs rapidly after synthesis of the fusion precursor and that degradation of the mature protease is proportionally correlated with its activity in this system.

### Autoprocessing of GST fusion precursors does not require TFR

**Figure 5:**
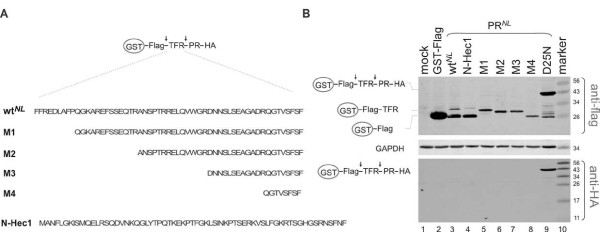
**The TFR is dispensable for autoprocessing of the fusion precursors**. **A**. Schematic diagram depicting truncation and replacement (N-Hec1) of TRF amino acid sequences in the GST-Flag-TFR-PR*^NL ^*precursor. **B**. Autoprocessing of the resulting fusion precursors in transfected HEK293T cells. Post-nuclear cell lysates were prepared at 40 h post transfection and subjected to western blotting using monoclonal mouse anti-Flag or anti-HA primary antibodies and IR800 goat anti-mouse second antibody. Schematic diagrams of the full length fusion precursor and processing products are indicated at left. Molecular mass markers (kDa) are indicated at right.

Recently, Leiherer et al reported that the TFR region is dispensable after it is uncoupled from the p6 coding sequence in a NL4-3-derived proviral context. For comparison, we here sought to examine TFR function in the context of the GST fusion precursors to further evaluate the system. A series of N-terminal TFR truncations in the context of GST-TFR-PR*^NL^*-HA backbone were constructed; the shortest TFR mutant only has eight residues upstream of the proximal cleavage site (Figure 5A). Interestingly, all of the TFR truncation precursors were capable of autoprocessing; no full-length precursor was detected. For the wild type precursor, the distal cleavage was more favourable. However, in the absence of the distal cleavage site, as in the N-terminal truncations, effective autoprocessing was also observed, indicating flexibility of cleavage site usage during autoprocessing. We also constructed a mutant in which the TFR was replaced with an unrelated peptide (N-Hec1; 66 residues derived from the N-terminus of the Hec1 protein) while keeping the last four residues upstream of the proximal cleavage site. The resulting fusion precursor also autoprocessed as efficiently as the wild type control (Figure 5B lanes 3 & 4). Collectively, our data demonstrated that the majority of the TFR was dispensable for autoprocessing of fusion precursor in transfected mammalian cells.

## Conclusions and Discussion

It has long been believed that HIV protease autoprocessing is a highly regulated reaction concomitant with virion release. However, the detailed molecular and cellular mechanisms of autoprocessing regulation remain poorly understood. This is partially attributed to lack of appropriate model systems for the study. Most HIV protease studies have been structure based - there are about four hundred protease structures reported in the literature. Almost all crystallized structures are for the dimeric mature protease, the final product of autoprocessing. In contrast, no structural information for the protease precursor is available except for a single monomer protease structure that has been reported using NMR analysis of a modified pseudo wild type protease containing a four-residue extension from the N-terminus and a four-residue deletion of the C-terminus [8,23]. Structural analyses of mature protease dimers alone cannot fully explain the autoprocessing mechanism or reveal the cause of drug resistance. Proviral DNA mutagenesis on the other hand has provided insightful information regarding protease autoprocessing mechanism [14,19-22,24,28], however, sensitive and direct detection of the mature protease, along with its precursor and processing intermediates, has been restrained due to lack of highly specific and sensitive antibodies. Consequently, most information is indirectly derived from analysis of Gag processing efficiency and/or p24 production [14,24]. We here report a simple model system to examine protease autoprocessing in transfected mammalian cells, which allows detection of some processing products at the steady state in the cell lysate. Importantly, this system was able to reproduce previously reported phenotypes that were described using mutant provirus constructs, further validating its utility for autoprocessing analysis. Autoprocessing of the GST fusion precursors was also sensitive to protease inhibitors; this cell based system may be further developed for screening new drugs that inhibit HIV protease autoprocessing.

The GST fusion precursors also revealed some interesting properties of protease autoprocessing. First of all, fully active mature proteases were not detectable in the cell lysates, whereas some mutant proteases such as PR*^pse ^*H69E and PR^NL ^H69D with reduced activities were detected. We interpreted this to indicate that the fully active protease is rapidly degraded in transfected cells likely via autoproteolysis as it was reported many years ago [25]. Given that the mature protease recognizes a wide variety of substrate sequences, autoproteolysis of the mature protease might be advantageous to the virus once the protease has completed processing of Gag and Gag-Pol in the virion. With our mammalian expression system, the degradation efficiency was positively correlated with protease activity at steady state levels; more mature protease was detected when protease activity was decreased. Detection of the mature form of wild type protease in the presence of protease inhibitors is consistent with this speculation. However, it remains to be defined whether additional mechanisms in mammalian cells are also attributed to the disappearance of fully active mature protease. It is worth noting that the mature pseudo wild type protease is engineered to be proteolysis resistant and is readily detectable in *E. coli *lysate. However, both the pseudo wild type and the NL4-3 derived wild type mature proteases are undetectable in transfected HEK293T cells. It is known that the concentration of PR plays a role in its autoproteolysis, so one possible explanation is that the steady state PR concentration in *E. coli *is different from that in mammalian cells, which remains to be determined.

Curiously, the present study also demonstrated that autoprocessing of the GST fusion precursor did not require fully active protease. Except for the D25N negative control, all the mutant fusion precursors demonstrated detection of the products generated following cleavage of the distal or proximal sites, respectively, in the cell lysates. Among them, PR*^pse ^*H69E is known to have reduced catalytic activity *in vitro *[24]; the observed accumulation of PR*^pse ^*H69E mature protease in the cell lysate was consistent with a reduced proteolytic activity. Nevertheless, the PR*^pse ^*H69E precursor generated similar amounts of processing products as the wild type controls, suggesting that PR*^pse ^*H69E is competent for autoprocessing of the GST fusion precursor even though it possesses reduced activity. The PR^NL ^H69D mutant also demonstrated a phenotype very similar to PR*^pse ^*H69E. These results appeared different from a previous report indicating that in VLPs produced by PR*^pse ^*H69E and PR^NL ^H69D proviruses, the full-length protease precursor is the predominant polypeptide and processed intermediates are undetectable[14,24]. We speculate this discrepancy to indicate that a suppressing mechanism exists to prevent Gag-Pol precursor autoprocessing in the context of the provirus, which is missing in the GST fusion precursor system. In the context of the provirus, a combination of reduced catalytic activity and a viable suppressing mechanism could completely abolish PR*^pse ^*H69E and PR^NL ^H69D autoprocessing. In the absence of the suppressing mechanism, as in the GST fusion precursors reported herein, the proteases with reduced activities were still able to autoprocess releasing mature protease. Further investigation is essential to define the suppressing mechanism.

Cleavage preference between the two authentic cleavage sites was also observed in this report. The proximal cleavage site was preferentially processed by the PR*^pse ^*precursor whereas the PR*^NL ^*precursor cleaved the distal site more frequently. A simple interpretation would be that the two proteases have different substrate preferences because amino acid sequences at the cleavage sites are identical in the two precursors. A previous study demonstrated that both sites are cleaved at similar rates by mature protease when added *in trans *to the HIV-1 Gag-PR^D25A^-Pol precursor in an *in vitro *assay [13]. At low concentrations (~ 0.2 nM), the HIV-1 Gag-Pol precursor preferentially processed the distal site [6]. Therefore, the result of our PR*^NL ^*precursor is consistent with the previous reports further validating it as a model system for HIV autoprocessing studies. The PR*^pse ^*precursor might fold into a structure different from the PR*^NL ^*precursor due to different protease sequences (six substitutions [14]), resulting in different substrate exposure. Alteration of H69 to other amino acids (Q and E) in the PR*^pse ^*backbone changed cleavage preference, also supporting the latter idea. Nevertheless, detailed structural analysis of these precursors will be required to determine the mechanism of cleavage preference.

The role of the TFR in protease autoprocessing has been difficult to assess because the coding sequence overlaps with the frameshifting signal and the p6 coding sequence. Using our GST fusion system, we demonstrated that the TFR is not required for the proximal cleavage event that releases mature protease. Additionally, replacement of the TFR with another unrelated peptide (N-Hec1) did not impact autoprocessing. This result is consistent with a recent report by the Ralf Wagner group demonstrating that partial substitution or deletion of 63% of the TFR did not affect virus growth and infectivity [28]. Collectively, these data suggest that TFR mainly serves as a linker between the frameshift site and the mature protease. Consistent with this role, the TFR polypeptide has not been shown to have a defined structure by itself. However, it remains to be determined whether TFR regulates protease structures in an auxiliary manner during autoprocessing in the infected cell. In line with this, about two decades ago Partin et al reported that TFR deletion enhances the proteolytic processing of an HIV-1 protease precursor generated by *in vitro *transcription/translation [29]. Therefore, more investigation will be necessary to further define TFR function.

## Methods

### DNA mutagenesis

Plasmids used in this study were generated following standard molecular cloning procedures. Construction of pGEX-3X-derived plasmids expressing GST-TFR-PR*^pse^*-Flag and GST-TFR-PR*^D25N^*-Flag and construction of NL4-3-derived Gag-PR*^pse ^*and Gag-PR*^D25N ^*proviruses were described previously [24]. All plasmids for mammalian expression of GST-fused miniprecursors were derived from the pEBG parental vector in which expression of GST is driven by the human EF-1α promoter [30]. The TFR sequence was derived from NL4-3 and the protease sequences were either from NL4-3 or a previously described pseudo wild-type protease [24]. In order to facilitate detection of the full length precursor and its derivatives, sequence encoding a Flag tag was inserted between the GST and TFR coding sequences and sequence encoding a HA tag was added to the C-terminus of the PR coding sequence. Mutations were introduced into the GST-Flag-TFR-PR-HA backbone by PCR-mediated site-directed mutagenesis. Template plasmid encoding the N-terminus of Hec1 (Highly expressed in cancer) [31] was kindly provided by Dr. Jennifer Deluca (Colorado State University) for PCR amplification of the insert. All the plasmids were verified by DNA sequencing and the sequence information is available upon request.

### Bacterial expression of GST fused miniprecursors

The pGEX-3X-derived plasmids were transformed into *E. coli *BL21 (Novagen, San Diego, CA), and transformed colonies were individually grown in Luria-Bertani medium at 37°C overnight. The overnight culture was then diluted 100-fold into 2×YT medium (10 g/L yeast extract, 16 g/L tryptone, 5 g/L NaCl) and incubated at 37°C for 2.5~3 h prior to the addition of isopropyl thiogalactoside (IPTG; 40 μM) to induce protein expression. Following addition of IPTG, cells were incubated for 4 h at 30°C and then cells were collected by centrifugation. For western blot analysis, cell pellets derived from equal volumes of culture medium were directly lysed in SDS/PAGE loading buffer.

### Cell culture, transfection and western blotting

Human embryonic kidney-derived 293T cells were maintained in DMEM (Dulbecco's Modified Eagle's Medium; Invitrogen, Carlsbad, CA) as previously described [24,32]. For *in vitro *transfection, 293T cells were plated in 6-well plates and incubated overnight to achieve 50-60% confluence at the time of transfection. One hour prior to transfection, chloroquine was added to each well to a final concentration of 25 μM. A total of 1 μg DNA in 131.4 μL of ddH_2_O was mixed with 18.6 μl 2 M CaCl_2 _to give a final volume of 150 μl. Then, 150 μl of HBS (50 mM HEPES, 280 mM NaCl, 10 mM KCl, 12 mM Dextrose, 1.5 mM Na_2_HPO_4_, pH 7.05) was added drop-wise to the DNA solution. The resulting mixture was directly added to the 293T cells. After 7-11 h of incubation, the culture medium was replaced with chloroquine-free DMEM.

To examine proteins in transfected cells, post-nuclear cell lysates were prepared as described previously [24,32]. In brief, transfected cells from each well of a 6-well plate were lysed in situ using 200 μL lysis buffer (25 mM Tris-HCl pH 8.0, 150 mM NaCl, 1% sodium deoxycholate, 1% Triton X-100, and protease inhibitor cocktail). The lysate was then centrifuged at 20800× *g *for 2 min to remove the nuclei and 20 μL of the resulting supernatant was subjected to SDS-PAGE followed by western blot analysis using polyvinylidene fluoride membrane. Unless indicated otherwise, cell lysates were prepared 40-48 h post transfection. To examine proteins associated with released virus-like particles (VLPs), culture medium was collected 11-48 h post transfection and centrifuged at 20,800 × *g *for 2 min at ambient temperature. The clarified supernatant was then collected and centrifuged at 20,800 × *g *for 3 h at 4°C to pellet virions. Virion pellets were resuspended in 30 μL PBS and 15 μL aliquots were subjected to SDS-PAGE analysis.

Mouse anti-HIV p24 (Cat# 3537) and rabbit anti-HIV-1 protease serum (Cat# 4105) were obtained from the NIH AIDS research and reference program. Purchased primary antibodies included mouse anti-HA, anti-FLAG, (Sigma, St. Louis, MO) and mouse anti-GAPDH (Glyceraldehyde-3-phosphate dehydrogenase; clone 6C5, Fisher Scientific, Pittsburgh, PA). Polyclonal rabbit anti-GST, a kind gift from Dr. Santiago Di Pietro (Colorado State University), was raised against purified GST-Rab38 and GST-Rab32 proteins and purified through GST column. Infrared dye-labeled secondary antibodies were obtained from Rockland Immunochemicals, Inc. (Gilbertsville, PA). Western blot images were captured using an Odyssey infrared dual laser scanning unit (LI-COR Biotechnology, Lincoln, Nebraska).

## Competing interests

The authors declare that they have no competing interests.

## Authors' contributions

CC designed the experiments and wrote the manuscript. LH performed all the experiments. Both authors read and approved the final manuscript.
